# How antiretroviral therapy (ART) programmes in Africa can maintain treatment for people living with HIV (PLHIV) during COVID-19 pandemic

**DOI:** 10.11604/pamj.supp.2020.35.2.24180

**Published:** 2020-06-21

**Authors:** Cavin Epie Bekolo

**Affiliations:** 1 The University of Dschang Taskforce for the Elimination of Covid-19 (UNITED#Covid-19), Dschang, Cameroon; 2 Department of Public Health, University of Dschang, Dschang, Cameroon; 3 Research Reaching Rural & Remote Residents (5R), District Health Service, Nkongsamba, Cameroon

**Keywords:** HIV, ART, retention, differentiated care, COVID-19, Africa

## To the Editors of the Panafrican Medical Journal

Remarkable progress has been achieved in the past decade with record numbers accessing antiretroviral therapy (ART) in developing countries at the epicentre of the HIV epidemic in sub-Saharan Africa. However, the momentum of scaling up access to ART and maintaining the rising number of PLHIV on treatment is facing a significant threat from the rapid spread of the coronavirus pandemic to and within these countries. We learnt from the Ebola outbreak in 2014-2015 that a significant decrease in health service utilisation for HIV care was reported with rates of defaulting in ART care reaching 42% at the peak of the epidemic in Guinea [1-3]. The already fragile health systems in SSA are yet to be further stretched given fewer cases of COVID-19 reported so far but the anxiety surrounding the virus coupled with restrictive measures to contained it suggests that PLHIV and healthcare workers are ‘staying at home’ for fear of contracting the virus in the healthcare setting. The likely consequence is that PLHIV may die mostly from interruptions in their ART care than from COVID-19. In responding to COVID-19 countries in SSA must think about sustaining essential services for prevailing conditions including HIV-AIDS which is a more deadly disease than COVID-19 in the continent. There is no one way of doing this because of context-specific challenges.

Some of the ways by which we can keep patients on ART while they respect the “stay-at-home” messages are: multi-month prescriptions (MMP) and Multi-Month Dispensing (MMD) for at least 3 months and up to 6 months especially for stable patients [4,5]. This allows for ample and interrupted medication supply for patients and reduction of workload for healthcare workers. This may however require a strong supply chain management system for antiretroviral medicines to avoid stockouts that are unfortunately frequent; appointment spacing for clinical visits to at least 3 months and up to 6 months for stable patients [6]. Less frequent contacts between HCW and patients will reduce potential exposure to SARS-CoV-2 for both and will ease pressure on the already stretched health system; community ART distribution points managed by PLHIV themselves who form a network and are trained to provide ART refills, adherence support and follow-up of basic support and follow-up health assessments as in Kinshasa in the Democratic Republic of the Congo [5].

Community ART groups (CAGs): they are self-formed groups of about six stable patients on ART from a community in the same geographic location as in Tete in Mozambique. Group members take turns every 3 - 6 months collecting antiretroviral medicines at the clinic for all the members and then redistribute medication to other group members. Members also provide adherence support and monitor treatment outcomes [5]; home delivery and dispensation of medicines by community health workers who equally provide psychological and social support for PLHIV as practised in Cameroon; virtual support for PLHIV who need clinical care via telephone, messaging including whatsApp, telemedicine, online portals and other available digital tools to minimise the need to access an overburdened health system during time of response and risk increased exposure to SARS-CoV-2 at health facilities; health facilities should develop specific standard operating procedures with clear patient routes and specific infection prevention and control (IPC) measures to ensure safety for personnel and patients including PLHIV who need clinical consultations.

## Conclusion

We hope these models of care delivery that have demonstrated their potential to reduce burdens for patients and the health system, to increased retention in care and to lower service provider costs in normal and emergency situations, may be used in any combination according to context to ensure uninterrupted ART care in SSA as the pandemic unfolds.

## Competing interests

The authors declare no competing interests.
